# Covariates of intravenous paracetamol pharmacokinetics in adults

**DOI:** 10.1186/1471-2253-14-77

**Published:** 2014-09-13

**Authors:** Karel Allegaert, Klaus T Olkkola, Katie H Owens, Marc Van de Velde, Monique M de Maat, Brian J Anderson

**Affiliations:** 1 Department of Development and Regeneration, KU Leuven, Leuven, Belgium; 2 Neonatal Intensive Care Unit, University Hospitals Leuven, Herestraat 49, Leuven, Belgium; 3 Department of Anaesthesiology, Intensive Care, Emergency Care and Pain Medicine University of Helsinki and Helsinki University Central Hospital, Helsinki, Finland; 4 School of Pharmacy, University of Otago, Dunedin, New Zealand; 5 Department of Cardiovascular Sciences, KU Leuven, Leuven, Belgium; 6 Department of Anaesthesiology, University Hospital Leuven, Leuven, Belgium; 7 Department of Clinical Pharmacy, Rijnstate Hospital Arnhem, Arnhem, The Netherlands; 8 Department of Anaesthesiology, University of Auckland, Auckland, New Zealand

## Abstract

**Background:**

Pharmacokinetic estimates for intravenous paracetamol in individual adult cohorts are different to a certain extent, and understanding the covariates of these differences may guide dose individualization. In order to assess covariate effects of intravenous paracetamol disposition in adults, pharmacokinetic data on discrete studies were pooled.

**Methods:**

This pooled analysis was based on 7 studies, resulting in 2755 time-concentration observations in 189 adults (mean age 46 SD 23 years; weight 73 SD 13 kg) given intravenous paracetamol. The effects of size, age, pregnancy and other clinical settings (intensive care, high dependency, orthopaedic or abdominal surgery) on clearance and volume of distribution were explored using non-linear mixed effects models.

**Results:**

Paracetamol disposition was best described using normal fat mass (NFM) with allometric scaling as a size descriptor. A three-compartment linear disposition model revealed that the population parameter estimates (between subject variability,%) were central volume (V1) 24.6 (55.5%) L/70 kg with peripheral volumes of distribution V2 23.1 (49.6%) L/70 kg and V3 30.6 (78.9%) L/70 kg. Clearance (CL) was 16.7 (24.6%) L/h/70 kg and inter-compartment clearances were Q2 67.3 (25.7%) L/h/70 kg and Q3 2.04 (71.3%) L/h/70 kg. Clearance and V2 decreased only slightly with age. Sex differences in clearance were minor and of no significance. Clearance, relative to median values, was increased during pregnancy (F_PREG_ = 1.14) and decreased during abdominal surgery (F_ABDCL_ = 0.715). Patients undergoing orthopaedic surgery had a reduced V2 (F_ORTHOV_ = 0.649), while those in intensive care had increased V2 (F_ICV_ = 1.51).

**Conclusions:**

Size and age are important covariates for paracetamol pharmacokinetics explaining approximately 40% of clearance and V2 variability. Dose individualization in adult subpopulations would achieve little benefit in the scenarios explored.

## Background

Paracetamol (*acetaminophen*) is the most commonly used drug to treat fever or pain, both as an over the counter drug as well as in the hospital setting [1]. Paracetamol can be administered either in monotherapy or as part of a multimodal approach, resulting in more effective temperature control when combined with non-steroidal anti-inflammatory drugs (NSAIDS) or equivalent analgesia with lower opioid exposure [2-4]. In healthy adults and using on label doses, paracetamol is almost exclusively eliminated by conjugation into either paracetamol glucuronide (47 - 62%) or paracetamol sulphate (25 - 36%), while limited amounts (1 - 4%) are excreted in the urine as unchanged paracetamol or undergo (<10%) oxidation to result in toxic metabolites (N-acetyl-p-benzoquinone, NAPQI) [5,6]. At higher doses, or in specific settings like alcohol abuse or malnutrition, the oxidative pathway may be more active and may result in hepatic necrosis [7]. When used in therapeutic dosages, paracetamol is generally regarded as safe and well tolerated in a variety of patients.

While oral and rectal formulations have been popular for the past century, an intravenous formulation has recently been introduced into clinical care. Such an intravenous formulation can be considered in the immediate postoperative period if the oral route cannot yet be used, while avoiding the unpredictability of absorption and bioavailability following rectal administration. The development of intravenous formulations has allowed time-concentration profile observations unencumbered by absorption variability. In addition to observations in healthy volunteers [8,9], the pharmacokinetics in special populations have been reassessed, including geriatric patients, abdominal surgery cases, intensive care patients and women at delivery or in postpartum [10-13].

Pooling of such datasets has the potential to further explore covariates, including weight, gender or disease characteristics [14-16]. Such an effort is of relevance. This is because a unique and single dosing regimen in any adult (i.e. 1 g intravenous paracetamol, q6h for maximal 48 hours) irrespective of other covariates may be an over-simplification, omitting clinical settings with either higher (insufficient effect) or lower clearance (raised risk for toxicity). Information on covariates of intravenous paracetamol disposition may be extrapolated to other routes of administration, or even to other compounds that undergo similar routes of elimination [5,17-19]. The current pooled intravenous paracetamol PK study explores the impact of covariates (e.g. age, weight, pregnancy, intensive care, type of surgery) on paracetamol disposition when compared to similar observations in healthy adult volunteers.

## Methods

### Clinical observations

**Figure 1:**
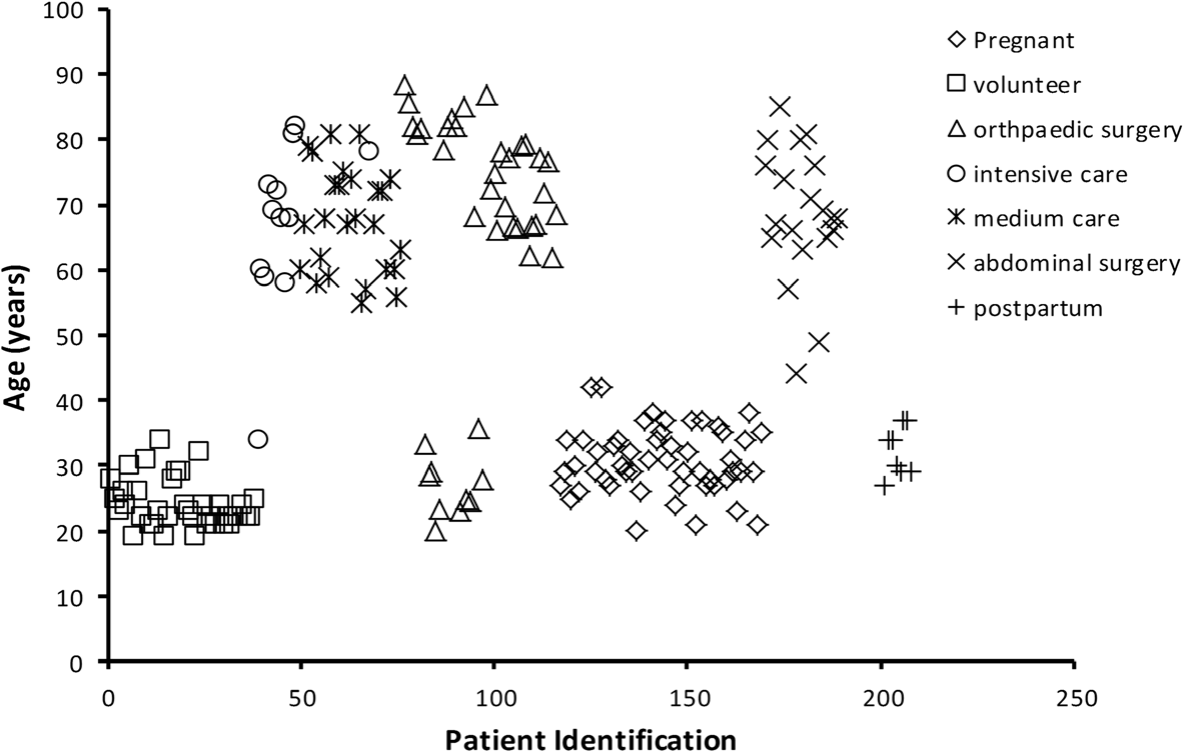
Age distribution of included adults (volunteers or patients).

Observations of intravenous paracetamol disposition in different cohorts of adults published in the literature were pooled to explore covariate influences (e.g., gender, age, size, disease characteristics, surgical procedure). Cohorts were retrieved using a PubMed search that included the ‘snowball method’ , followed by an invitation to the corresponding authors to provide the raw data (time concentration profiles, clinical characteristics) within a setting of academic collaboration [20]. Patient demographics and age distribution are presented in Table 1 and Figure 1 respectively.

**Table 1:** Summary data of the pooled studies

**Reference**	***ref 8***	***ref 9***	***ref 11***	***ref 10***	***ref 12***	***ref 13***	***ref 11***	***ref 11***	***Ref 11***
**Characteristics**	12 healthy males	12 healthy males	8 healthy females	Orthopaedic surgery	High dependency and IC, 38 cases	Abdominal surgery	Caesarean delivery	Postpartum early	Postpartum late,
		14 healthy females		40 cases		20 cases	41 cases	8 cases (15 weeks)	7 cases (1 year)
**Study design**	Single iv bolus, 0.5 g	iv loading dose 2 g,	single iv bolus, 2 g	Single iv bolus, 1 g	Single iv bolus, 1 g	repeated 1 g, 6 qh	iv loading dose 2 g, maintenance 1 g q6h	iv loading 2 g	iv loading 2 g
		Maintenance 1 g q6h				48–72 h			
**Weight**	63-83 kg	49-94 kg	54-74 kg	58-107 kg	53-120 kg	57-101 kg	57-110 kg	52-88 kg	50-87 kg
**Age**	21-25 years	19-34 years	27-37 years	20-88 years	34-82 years	44-85 years	31, SD 5.8 years	31, SD 5.8 years	32, SD 6 years
**Sampling strategy**	13 samples/case	32 samples/case	4 samples/case	20 samples/case	8 samples/case	2x9 samples/case	4 samples after loading dose + up to 24 h	4 samples/case	4 samples/case,
	up to 24 h	up to 48 h	up to 6 h	up to 24 h	up to 6 h	up to 72 h		up to 6 h	up to 6 h
**Analytical technique**	HPLC-uv	HPLC-uv	HPLC-uv	HPLC	Fluorescent polarization immuno-assay	HPLC	HPLC-uv	HPLC-uv	HPLC-uv
**LLOQ**	< 0.1 μg/ml	< 0.02 μg/ml	< 0.08 μg/ml	< 0.25 μg/ml	< 0.2 μg/ml	n.a.	< 0.08 μg/ml	< 0.08 μg/ml	< 0.08 μg/ml
**CV%**	<6.4%	< 3%	< 15%	< 12.8%	< 7.5%	n.a.	< 15%	< 15%	< 15%

[IC = intensive care; iv = intravenous; SD = standard deviation, HPLC = High Pressure Liquid Chromatography; LLQ = lower limit of quantification; CV = coefficients of variation].

#### Healthy volunteer studies

Twelve healthy male volunteers (21–25 year, 63–83 kg) were given a single dose of intravenous (IV) propacetamol (1 g, equal to 0.5 g of paracetamol, Pro-Dalfagan, Bristol-Myers Squibb Pharmaceuticals, Braine l′Alleud, Belgium) and 13 blood samples for assay were subsequently collected for up to 24 h afterwards [8]. Paracetamol concentrations were quantified in plasma by reverse High Pressure Liquid Chromatography (HPLC) with UV detection. The lower limit of quantification was 0.1 μg/ml. Intra-assay coefficients of variation (CV) at 0.04, 1.25 and 5 μg/ml were 6.4, 1.9 and 2% respectively, inter-assay CV at 0.5 and 10 μg/ml were 3.3 and 2.2% respectively.

Healthy male (n = 12) and female (n = 14) volunteers (19–34 year, 49–94 kg) were given IV paracetamol (2 g loading dose, followed 1 g intravenous paracetamol 6 hourly, Perfalgan, Bristol Myers Squibb, Paris, France) [9]. Plasma samples (n = 32) were collected for up to 48 h after the loading dose were collected, with specific emphasis after the first and after the final 5^th^ paracetamol dose (at 24 h). All female volunteers were on contraceptives during the study, 13/14 based on oral contraceptives. Paracetamol concentrations were quantified in plasma by reverse HPLC with UV detection. The lower limit of quantification was 0.02 μg/ml. Imprecision and inaccuracy were lower than 3% and within 1% respectively.

Eight healthy female volunteers (27–37 year, 54–74 kg) were studied following a single loading dose (2 g IV paracetamol, Perfalgan, Bristol Myers Squibb, Braine l′Alleud, Belgium or intravenous Paracetamol, Fresenius Kabi, Schelle, Belgium) (1,2,4,6 h) as part of a research project concerning intravenous paracetamol disposition in pregnancy and postpartum [11]. None of these volunteers were on oral contraceptives. Paracetamol concentrations were quantified in plasma by reverse HPLC with UV detection. The lower limit of quantification was 0.08 μg/ml. Coefficients of variation for intra- and inter-day precision and accuracy were all below 15%.

#### Clinical cohorts

Single dose IV paracetamol (1 g, Perfalgan 10 mg/L solution, Bristol-Myers Squibb, Agen, France) pharmacokinetics have been documented in 40 patients following orthopaedic surgery, with a study design to explore the age related impact (20–88 year, 58–107 kg, male/female = 19/21) [10]. Plasma samples (n = 20) were collected in each patient for up to 24 h. Paracetamol plasma concentrations were quantified in plasma by HPLC. The lower limit of quantification was 0.25 μg/ml. The interday CV for paracetamol was 12.8, 12.5 and 5.1% at 0.398, 2.01 and 10.1 μg/ml respectively.

As part of a study on IV paracetamol tolerance during repeated administration in adults admitted in medium (high dependency) and intensive care, paracetamol concentrations were quantified in 38 medium and intensive care patients (34–82 year, 53–120 kg, male/female = 27/11) after the first administration (1 g, Perfalgan, Bristol-Myers Squibb BV, Woerden, The Netherlands) [12]. Blood samples were collected up to 6 h after initiation of intravenous administration with a ‘trough’ concentration recorded before the second administration. Paracetamol serum concentrations were quantified with fluorescent polarization immunoassay (Cobas Integra 400, Roche Diagnostics, West Sussex, UK). Lower limit of detection of the analysis was 0.2 μg/ml. Within-run variation and total variation for low as well as high concentrations (9.9, 32.9 and 97.4 μg/ml) were within a range of 0.7-5.8% and 4.4-7.5% respectively.

Twenty patients received IV paracetamol (1 g, 6 hourly, up to 48–72 h, Perfalgan Bristol-Myers Squibb Ltd, Auckland, New Zealand) after major abdominal surgery (44–85 year, 57–101 kg, male/female = 8/12) [13]. Plasma samples were collected over 2 intervals (day of surgery and 2–3 days afterwards). Paracetamol concentrations were quantified by HPLC.

Repeated dose IV paracetamol pharmacokinetics (loading dose 2 g, followed by 1 g 6 hourly for 24 h) were collected in a cohort of 41 women undergoing caesarean delivery [11]. A subgroup of 8/41 women initially included at delivery were recruited for a second single loading dose (2 g paracetamol) PK study 10–15 weeks after delivery and 7/8 women were re-evaluated a third time (single loading dose, 2 g) about one year after delivery [11]. Blood samples were collected after the loading dose (1, 2, 4 h) with subsequent collection at trough (6, 12, 18 and 24 h). More recently, 8 additional observations in women undergoing caesarean delivery were collected, resulting in 49 observations at delivery. Paracetamol plasma concentrations were determined by HPLC. The lower limit of quantification was 0.08 μg/ml. Coefficients of variation for intra- and inter-day precision and accuracy were all below 15%.

#### Pharmacokinetic analysis

Population parameter estimates were obtained using non-linear mixed effects modeling (NONMEM 7.3, Globomax LLC, Hanover, MD, USA). This software accounts for population parameter variability (between subjects) and residual variability (random effects) as well as parameter differences predicted by covariates (fixed effects). The population parameter variability (or between subject variability, BSV) for structural model parameters were assumed to be log-normally distributed across the population.

$$\mathrm{CLi}=\mathrm{TVCL}\cdot e^{\mathrm{\eta CLbsv}+\mathrm{\eta CLbov}}$$

$$\mathrm{Vi}=\mathrm{TVV}e^{\mathrm{\eta Vbsv}+\mathrm{\eta Vboc}}$$

*η*CLbsv is the difference between individual (CLi) and population mean (TVCL), *η*CLbov is the difference in CL between occasions. *η*Vbsv is the difference between individual (Vi) and population mean (TVV), and *η*Vbov is the difference in V between occasions.

Residual unexplained variability (RUV) was modelled using additive and proportional terms. The variance of the RUV (η_RUV,i_) was also estimated.

$$\mathrm{Ci}=F\;e^{\mathrm{CVCP}}+\mathrm{SDCP}$$

Ci is to concentration in the individual, F is the model predicted concentration, CVCP is the coefficient of variation for the proportional error, and SDCP is the standard deviation of the additive error. Data from each assay laboratory was assigned individual.

The first order conditional interaction estimate method using ADVAN3 TRAN4 was used to estimate population mean parameters, between subject variance and residual variance. Convergence criterion was 3 significant digits.

Initial analyses suggested a three-compartment disposition model for paracetamol and the model was parameterized in terms of clearance (CL), inter-compartment clearances (Q2, Q3), central volume (V1) and peripheral volumes (V2, V3). The population parameter variability was modelled in terms of random effect (*η*) variables. Each of these variables was assumed to have mean 0 and a variance denoted by *ω*^2^, which was estimated. The covariance between two elements of *η* (e.g. CL and V) is a measure of statistical association between these two variables. Their covariance is related to their correlation (*R*) i.e.

$$R=\mathrm{covariance}/✓\left({w^{2}}_{\mathrm{CL}}\;x\;{w^{2}}_{V}\right)$$

The covariance of parameter variability was incorporated into the model.

#### Covariate analyses

a) *Size*

We investigated three measures of body size

Total body weight (TBW) (kg)

Fat Free Mass (FFM)

Fat free mass (FFM) can be predicted from TBW and height (H, m) [21].

$$\mathrm{FFM}=\mathrm{WH}S_{\text{max}}\cdot H^{2}\cdot \left[\mathrm{TBW}/\left(\mathrm{WH}S_{50}\cdot H^{2}+\mathrm{TBW}\right)\right]$$

where WHS_max_ is the maximum FFM for any given height (H, m) and WHS_50_ is the TBW value when FFM is half of WHS_max_. For men, WHS_max_ is 42.92 kg/m^2^ and WHS_50_ is 30.93 kg/m^2^ and for women WHS_max_ is 37.99 kg/m^2^ and WHS_50_ is 35.98 kg/m^2^.

normal fat mass (NFM)

Normal fat mass (NFM) is an extension of the concept of predicted normal weight [22] with a parameter (*Ffat*) which accounts for different contributions of fat mass (i.e. TBW minus FFM)

$$\mathrm{NFM}\left(\mathrm{kg}\right)=\mathrm{FFM}+\mathrm{Ffat}\cdot \left(\mathrm{TBW}-\mathrm{FFM}\right)$$

Instead of assuming a fixed value of *Ffat* in all cases the idea of NFM is to estimate the value of *Ffat* that is most appropriate for the parameter being predicted. If *Ffat* is estimated to be zero then FFM alone is required to predict size while if *Ffat* is 1 then size is predicted by TBW. Other estimates of *Ffat* reflect different weighting of body composition components.

The parameter values were standardised for a body size using an allometric model [23,24].

$$\mathrm{Pi}=\mathrm{Pstd}\times {\left(\mathrm{Xi}/\mathrm{Wstd}\right)}^{\mathrm{PWR}}$$

where *P*_*i*_ is the parameter in the i^th^ individual, *X*_*i*_ is a measure of body size (TBW, FFM or NFM) in the i^th^ individual and *P*_*std*_ is the parameter in an individual with a standard size *W*_*std*_ . The PWR exponent is 0.75 for clearance and 1 for distribution volumes [25-27]. Thus total drug clearance may be expected to scale with a power of ¾ with the allometric model:

$$\mathrm{CLi}=\mathrm{CLstd}\times {\left(X_{i}/70\right)}^{3/4}$$

where CLstd is the population estimates for CL.

b) *Age*

The effect of age (years) on clearance or distribution volumes was investigated using a scaling factor *(F*_*AGECL*_*, F*_*AGEV*_*).* The majority of patients were either younger than 40 years or older than 60 years (Figure 1). The formula for FFM was based on adults aged up to 60 years. Consequently if patients were aged above 60 years, then a scaling factor (F_AGE_) was applied to CL or V population estimates.

c) *Sex*

The male was taken as the standard and a scaling factor (F_SEXCL_) estimated if the patient was female:

$$\mathrm{CL}=F_{\mathrm{AGECL}}*F_{\mathrm{SIZECL}}*F_{\mathrm{SEXCL}}*\mathrm{CLstd}$$

d) *Other covariates*

A similar approach, using a scaling factor was taken with other covariates [pregnancy (F_PREG_), postpartum (F_PP_), intensive care (F_IC_), high dependency care (F_HD_) abdominal surgery (F_ABD_) and orthopaedic surgery (F_ORTHO_)] and their impact on clearance or volume respectively, e.g.

$$\begin{array}{ll} \mathrm{CL}= & F_{\mathrm{AGECL}}*F_{\mathrm{SIZECL}}*F_{\mathrm{SEXCL}}*\;F_{\mathrm{PREG}}*\;F_{\mathrm{ICCL}}\\ & *F_{\mathrm{HDCL}}*\;F_{\mathrm{ABDCL}}*\mathrm{CLstd} \end{array}$$

$$V2=F_{\mathrm{SIZEV}2}*F_{\mathrm{AGEV}2}*F_{\mathrm{ORTHOV}2}*F_{\mathrm{ICV}2}*V2\mathrm{std}$$

#### Quality of fit

The quality of fit of the pharmacokinetic model to the data was sought by NONMEM’s objective function and by visual examination of plots of observed *versus* predicted concentrations. Models were nested and an improvement in the objective function was referred to the Chi-squared distribution to assess significance e.g. an objective function change (OBJ) of 3.84 is significant at α = 0.05. An objective function change of 6.635 (p < 0.01) was used to determine covariate inclusion. Bootstrap methods provided a means to evaluate parameter uncertainty [28]. A total of 1000 replications were used to estimate parameter confidence intervals. A visual predictive check (VPC) [29], a modeling tool that estimates the concentration prediction intervals and graphically superimposes these intervals on observed concentrations after a standardized dose, was used to evaluate how well the model predicted the distribution of observed plasma concentrations. Simulation was performed using 1000 subjects with characteristics taken from the pooled population. For data such as these where covariates such as dose, size, sex, age, or pathology are different for each patient, we used a prediction corrected VPC (PC-VPC) [30].

#### Simulation

A simulation study was performed to investigate both concentration variability in a 25 year old 70 kg healthy adult volunteer given a standard dose of intravenous paracetamol 1 g 6 hourly for 36 h, and typical time-concentration profiles in a 68 year old 70 kg male in intensive care, a 25 year 70 kg pregnant woman in her third trimester, the same woman 2 months postpartum weighing 60 kg, and a 68 year old 70 kg male undergoing abdominal surgery. The drug was infused over 15 minutes. Pharmacokinetic parameter estimates and their variability from this current pooled study were used to predict individual time-concentration profiles.

## Results

The pooled analysis included 2755 paracetamol observations in 189 individuals. The clinical characteristics and medical conditions of the individual studies were already mentioned in the methods section, but the pooled dataset of adults had a mean weight of 73 kg (range 49.2 – 120 kg) and age 46 years (range 19–88.5 year). The distribution of ages was shown in Figure 1. All data were above the lower limit of quantification reported from each of the individual studies.

The model building process is shown in Table 2. A three compartment disposition model was better than a one or two-compartment model. Size scaling using NFM and allometry reduced the objective function more than either TBW or NFM. The estimate for Ffat_CL_ approached 1 (Ffat_CL_ = 0.989) and when this estimate was fixed at 1, the objective function change was small. Fixing Ffat_CL_ to 0 increased the objective function (ΔOBJ 19.238). We had concerns that pregnant women may require a further “correction factor” but this turned out to be unnecessary since there was no improvement in the objective function when applied to either clearance or volume of distribution. Both clearance and the peripheral volume of distribution V2 were reduced in the elderly, but when elderly patients undergoing abdominal surgery were accounted for, this reduction was no longer apparent. Sex differences in clearance were minor and of no significance. Clearance, relative to the population median, was increased during pregnancy (F_PREG_ = 1.14), and decreased during abdominal surgery (F_ABD_ =0.715). Clearance was not different in postpartum women. Patients undergoing orthopaedic surgery had a smaller V2 (F_ORTHO_ = 0.649) while those in intensive care had an increased V2 (F_ICV_ = 1.51). Once these covariate effects were established we were unable to determine any further effect attributable to sex. The final covariates of value to describe clearance were allometry using TBW, pregnancy and abdominal surgery

$$\mathrm{CL}=F_{\mathrm{SIZECL}}*\;F_{\mathrm{PREGCL}}*\;F_{\mathrm{ABDCL}}*\mathrm{CLstd}$$

**Table 2:** Key model building steps and associated objective function changes

**Basic model**		**OBJ**
1-compartment - no size scaling		8350.263
2-compartment - no size scaling		5804.790
3-compartment – no size scaling		5762.275
3-compartment – no size scaling + individual study centre RUV		5210.072
+BOV		5024.826
+BOV + allometric scaling FFM		4956.866
+BOV + allometric scaling TBW		4942.960
+BOV + allometric scaling NFM		4927.983
+BOV + allometric scaling NFM Ffat_CL_ = 1		4924.844
+BOV + allometric scaling NFM Ffat_CL_ = 0		4946.221
+BOV + allometric scaling NFM Ffat_CL_ = 1 + F_AGEV_		4908.588
+BOV + allometric scaling NFM Ffat_CL_ = 1 + F_AGEV_ + F_AGECL_		4901.989
+BOV + allometric scaling NFM Ffat_CL_ = 1 + F_AGEV_ + F_AGECL_ + F_SEXCL_		4900.663
+BOV + allometric scaling NFM Ffat_CL_ = 1 + F_AGEV_ + F_AGECL_ + F_PREGCL_	-F_SEXCL_	4893.125
+BOV + allometric scaling NFM Ffat_CL_ = 1 + F_AGEV_ + F_AGECL_ + F_PREGCL_ + F_ICCL_		4888.551
+BOV + allometric scaling NFM Ffat_CL_ = 1 + F_AGEV_ + F_AGECL_ + F_PREGCL_ + F_ICVOL_	-F_ICCL_	4876.427
+BOV + allometric scaling NFM Ffat_CL_ = 1 + F_AGEV_ + F_AGECL_ + F_PREGCL_ + F_ICV_ + F_HDUCL_		4875.677
+BOV + allometric scaling NFM Ffat_CL_ = 1 + F_AGEV_ + F_AGECL_ + F_PREGCL_ + F_ICV_ + F_ORTHOV_	-F_HDUCL_	4839.712
+BOV + allometric scaling NFM Ffat_CL_ = 1 + F_AGEV_ + F_AGECL_ + F_PREGCL_ + F_ICV_ + F_ORTHOV_ + F_ABDCL_		4827.174
+BOV + allometric scaling NFM Ffat_CL_ = 1 + F_AGEV_ + F_PREGCL_ + F_ICV_ + F_ORTHOV_ + F_ABDCL_	-F_AGECL_	4827.284

An OBJ decrease of 6.635 (p < 0.01) for these nested models was deemed significant for covariate inclusion.

[BOV = between occasion variability; RUV = residual unexplained variability; FFM = fat free mass; TBW = total body weight; NFM = normal fat mass; CL = clearance; abd = abdominal surgery; preg = pregnant; IC = intensive care; HD = high dependency care; ortho = orthopaedic surgery].

The final covariates of value to describe V2 were allometry using NFM, age, orthopaedic surgery and intensive care.

$$V2=F_{\mathrm{SIZEV}2}*F_{\mathrm{AGEV}2}*F_{\mathrm{ORTHOV}2}*F_{\mathrm{ICV}2}*V2\mathrm{std}$$

Parameter estimates for the final model are shown in Table 3. Residual unexplained additive and proportional errors for all 5 study sites were similar except for the additive error for the study investigating elderly orthopaedic patients (the centre for that study reported the highest lower limit of quantification). The residual additive errors were 0.09, 0.03, 1.1, 0.07, 0.02 mg/L for each centre. The proportional errors were 0.09, 0.06, 0.04, 0.09, 0.15 for the same centres with a η_RUV,i_ of 0.231. The correlation of between subject variability for structural parameters is shown in Table 4.

**Table 3:** Standardised intravenous paracetamol population pharmacokinetic parameter estimates

**Parameter**	**Estimate**	**BSV**	**BOV**	**95% CI**
CLstd (L.h^-1^.70 kg^-1^)	16.7	0.246	0.231	15.2, 17.8
F_PREGCL_	1.14	-	-	1.02, 1.27
Ffat_CL_	1 FIX	-	-	-
F_ABDCL_	0.715	-	-	0.548, 0.832
V1std (L.70 kg^-1^)	24.6	0.555	-	21.7, 27.1
Q2std (L.h^-1^.70 kg^-1^)	67.3	0.257	-	56.1, 79.7
V2std ( L.70 kg^-1^)	23.1	0.496	0.051	20.2, 26.1
Ffat_VOL_	0.778	-	-	0.503, 0.933
F_ORTHOV2_	0.649	-	-	0.485, 0.876
F_ICV2_	1.51	-	-	1.07, 2.90
Fage_V2_	0.838	-	-	0.702, 0.968
Q3	2.04	0.713	-	1.69, 2.81
V3	30.6	0.789	-	20.4, 64.3

(BSV is the between subject variability, CLstd = standardized clearance; preg = pregnancy; IC = intensive care; abd = abdominal surgery; ortho = orthopaedic surgery; BOV = between occasion variability; CI = confidence interval).

**Table 4:** The correlation of parameter between-subject variability

	**CL**	**V2**	**V1**	**Q2**	**Q3**	**V3**
**CL**	1					
**V2**	0.060	1				
**V1**	0.265	-0.734	1			
**Q2**	0.254	0.793	-0.170	1		
**Q3**	0.956	-0.134	0.432	0.136	1	
**V3**	0.379	-0.818	0.530	-0.750	0.544	1

[CL = clearance; V = distribution volume: Q = intercompartemental clearance].

**Figure 2:**
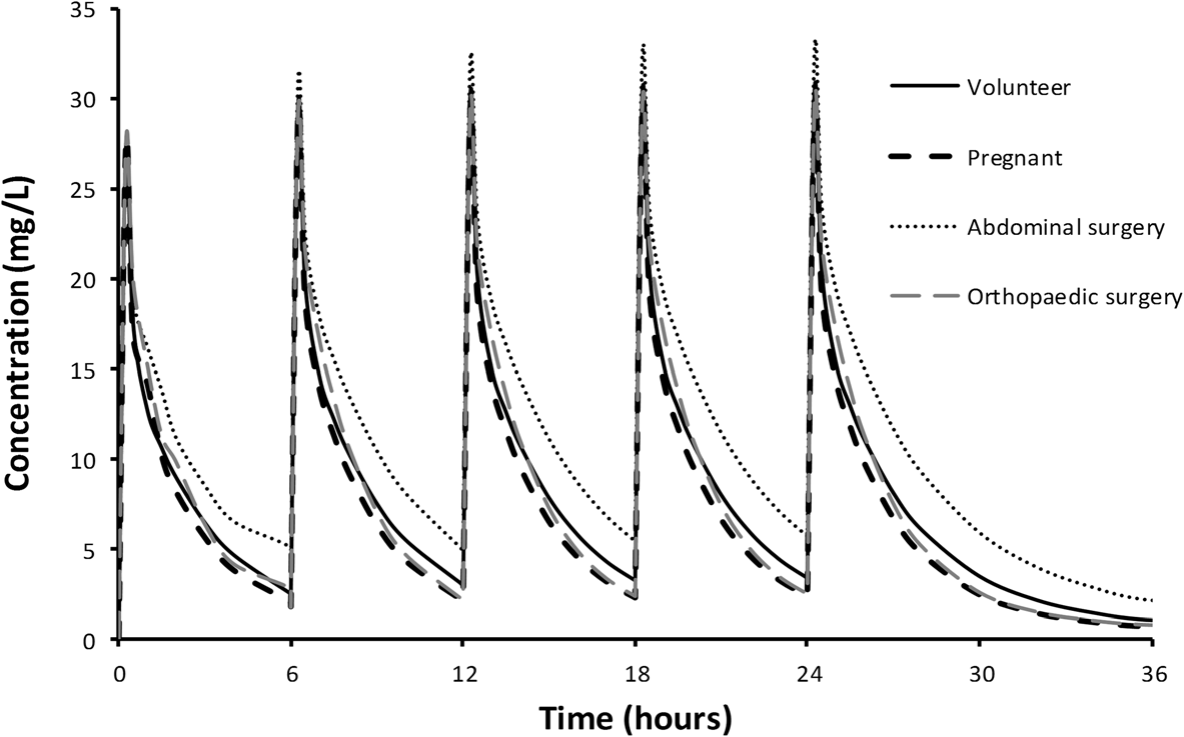
**Visual predictive check for the paracetamol 2-compartment model.** All plots show median and 90% intervals (solid and dashed lines). Left hand plot shows all observed concentrations. Right hand plot shows prediction percentiles (10%, 50%, and 90%) for observations (lines with symbols) and predictions (lines) with 95% confidence intervals for prediction percentiles (gray shaded areas).

**Figure 3:**
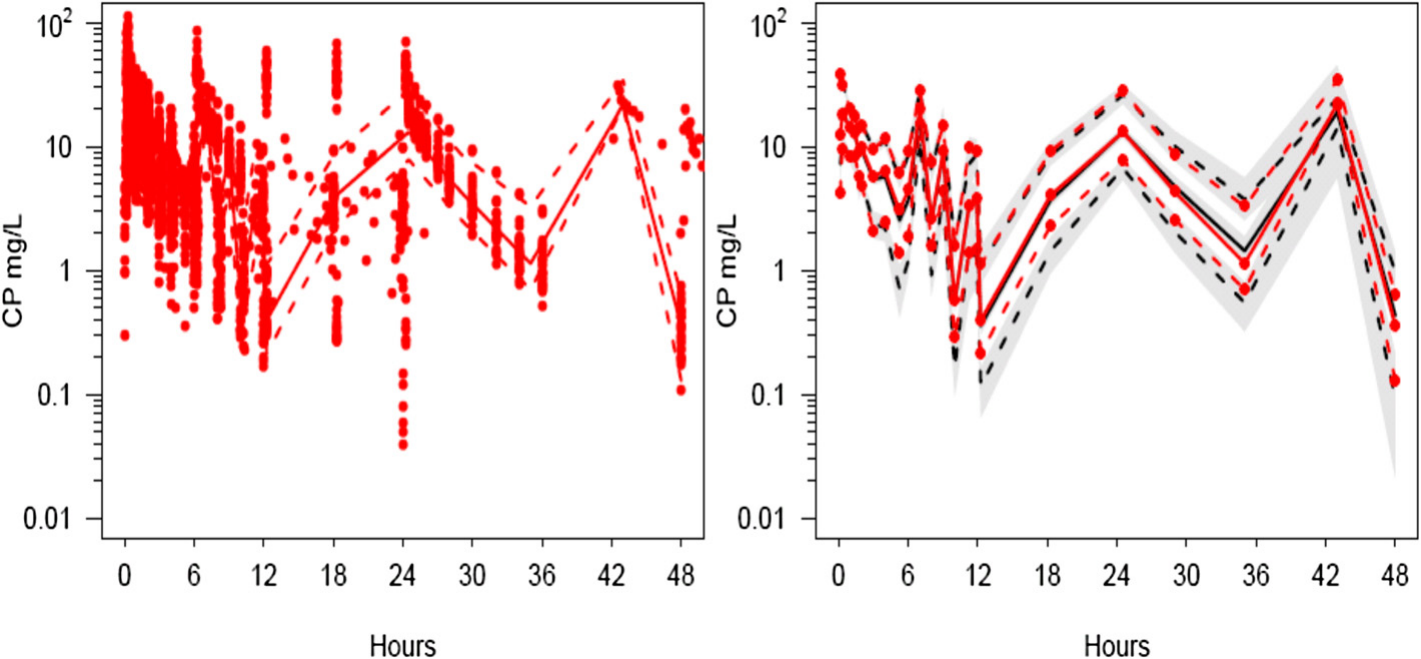
Time-concentration profiles for a 25 year old 70 kg healthy adult volunteer given a standard dose of intravenous paracetamol 1 g 6 hourly and typical time-concentration profiles a 25 year 70 kg pregnant woman in her third trimester and a 68 year old 70 kg male undergoing abdominal surgery.

Between occasion variability for clearance and V2 were 0.231 and 0.051 respectively. The between-subject variability (BSV) for clearance and V2 without covariates in the model were 0.436 and 0.666 respectively. This difference between BSV without covariates and with covariates is a measure of the predictable decrease in BSV due to covariates. The *ω*^2^ estimates for the different components contributing to variability of CL and V2 are shown in Tables 5 and 6 respectively. The ratio of the population parameter variability (PPVP^2^) predictable from covariates (BSVR^2^ + BOV^2^) to the total population parameter variability obtained without covariate analysis (PPVt^2^) gives an indication about how important covariate information is. For example, the ratio of 0.401 achieved for clearance in this current study indicates that 40.1% of the overall variability in clearance is predictable from covariate information.PC-VPC plots, used to demonstrate goodness of fit, are shown in Figure 2. Typical time-concentration profiles from the simulation study are shown in Figure 3. The mean concentration in patients with all types of pathology was 11.6 SD 1.9 mg/L. There is little difference in profiles due to the differences in pathology in this pooled analysis.

**Table 5:** **Effect of covariate analysis on variance (*ω***^**2**^**) of Clearance**

**Sequential nested model**	**PPVt**^**2**^	**BSVR**^**2**^	**BOV**^**2**^	**PPVP**^**2**^	**PPVP**^**2**^**/PPVt**^**2**^
**No covariates**	0.19	0.19	0	0	0
**Allometric scaling (TBW)**	0.19*	0.10	0.0413	0.0487	0.256
**Allometric scaling (NFM)**	0.19*	0.0889	0.0557	0.0454	0.239
**F**_**AGECL**_	0.19*	0.0799	0.0557	0.0544	0.286
**F**_**PREGCL**_	0.19*	0.0803	0.0513	0.0584	0.3074
**F**_**ABDCL**_	0.19*	0.0605	0.0533	0.0762	0.401

* = assumed from no covariate model estimate.

[PPV = population parameter variability; BSV = between subject variability; BOV = between occasion variability; TBW = total body weight; NFM = normal fat mass; preg = pregnant, abd = abdominal surgery; IC = intensive care patients; ortho = orthopaedic surgery].

**Table 6:** **Effect of covariate analysis on variance (*ω***^**2**^**) of V2**

**Sequential Nested Model**	**PPVt**^**2**^	**BSVR**^**2**^	**BOV**^**2**^	**PPVP**^**2**^	**PPVP**^**2**^**/PPVt**^**2**^
**No covariates**	0.437	0.437	0	0	0
**Allometric scaling (TBW)**	0.437*	0.396	0.00297	0.03803	0.087025
**Allometric scaling (NFM)**	0.437*	0.348	0.00325	0.08575	0.196224
**F**_**AGEV2**_	0.437*	0.304	0.00272	0.13028	0.298124
**F**_**ICV2**_	0.437*	0.299	0.00222	0.13578	0.310709
**F**_**ORTHOV2**_	0.437*	0.246	0.0258	0.1652	0.378032

* = assumed from no covariate model estimate.

## Discussion

Pooling of data allowed confirmation of the extent of the different covariates explored in individual studies and also provided new information about size scaling approaches. After allometric scaling and size standardization, pregnancy, and abdominal surgery, but not gender were significant covariates of clearance, explaining 40% of clearance variability. Age, intensive care and orthopaedic surgery in part (38%) explained the variability in distribution.

The paracetamol pharmacokinetic parameter estimates were the same as those predicted from paediatric data scaled using allometry with TBW [14]. Mature clearance, achieved within the first few years of life was 16.2 L/h/70 kg (BSV 0.45, BOV 23.5) and Vss was 63.2 L/70 kg. However, the best descriptor of size may not necessarily be total body weight, but rather may differ with each drug. Lean body mass (LBM) is appropriate for remifentanil, while propofol clearance in obese adults and non-obese adults and children is best predicted using TBW as the size descriptor with theory based allometry [31-34]. Paracetamol appears best described using normal fat mass (NFM) with allometric scaling as a size descriptor. This approach is versatile [34] because in addition to FFM (FFM is similar to LBW but excludes lipids in cell membranes and for all practical purposes these two descriptors are indistinguishable) there is an additional parameter, Ffat that characterizes the contribution of fat mass (TBW-FFM) to the apparent allometric size of the body. This parameter is drug specific (it depends on the physico-chemical characteristics of the compound) and also specific to the PK parameter such as clearance or volume of distribution [35].

A review of paracetamol pharmacokinetics as reported in literature [17,18,36-44] suggests that clearance decreases by 0.4%/year and volume of distribution decreases by 0.3%/year (using the young, 25 year old as the standard). This is equivalent to 20% decrease in CL and 15% decrease in volume of distribution from age 25 to age 75. The age distribution in this current study did not facilitate the use of a linear or exponential function to investigate this change. Although we noted a 12% decrease in V2 in the elderly, the contribution that age made to clearance was overshadowed by the reduced clearance noted in the elderly cohort who had abdominal surgery. These patients comprised an older cohort (age 67, range 49–85 years), and the severity of illness or frailty may have further contributed to reduced clearance. Wynne reports a further large decrease (36%) in clearance in frail elderly compared to healthy elderly [44]. A similar explanation may apply to the elderly cohort undergoing orthopaedic surgery who had a reduced volume of distribution. Volume changes probably reflect increased fat per kilogram body weight in the elderly, together with incomplete distribution of this non-lipophilic drug into body fat. Increased paracetamol clearance was observed during late trimester pregnancy, even after size scaling.

Others have reported an apparent oral clearance 58% higher in pregnant women compared to non-pregnant women [45,46]. After allowing for allometry and size models, we report a smaller increase in clearance than this estimate. The higher clearance in pregnant women (F_PREGCL_ = 1.14) is due to a higher than proportional increase in glucuronidation, a proportional increase in oxidation and a *sub*proportional increase in primary renal elimination [11]. Potentially hepatotoxic metabolites were not quantified in the maternal serum [11,46]. This increased clearance was no longer present 2–3 months after delivery when clearance was indistinguishable from the population mean [11].

There are data suggesting that women taking steroid oral contraceptives have increased glucuronidation of paracetamol of up to 50% and the impact of both pregnancy and oral contraceptives on intravenous paracetamol disposition [47,48]. Has recently been confirmed and may be driven by oestradiol [49]. In the current pooled analysis, we were unable to show that sex was a covariate.

Figure 2 demonstrates to what extent these patient related covariates affect the time-concentration profiles when compared to a reference 25 year old healthy volunteer. Predictions for healthy volunteer are not different from plots of typical individual with pathology. The mean concentration of 11.6 mg/L across all groups is consistent with the assumed target concentration of 10 mg/L associated with pain reduction of pain reduction of 2.6/10 [50]. Little is known about pharmacodynamic covariate effects in adults. Despite identification of pharmacokinetic covariate influences, the unexplained parameter variability still remains high (60% for CL) and dose individualization or subpopulation ‘tailored’ dosing would achieve little benefit in the scenarios observed. Target concentration intervention would be of little value. It is of use if a response, such as blood pressure, is substitute for measuring the clinical disease state that is being treated. When the medicine is working well or it is not working at all the clinical disease state may appear to be the same. It is assumed that trying to reach a typical response that is usually associated with benefit is better than giving everyone the same dose. The second reason for using target concentration intervention is when group based dosing (e.g. using weight) is not enough to reduce the between subject variability so that the medicine can be used safely and effectively. Target concentration intervention can only work however if the within subject variability is small enough so that dose individualization is really predictive for future use of the medicine in the same patient. The clearance covariate analysis on variance (*ω*^2^) only accounts for 40% of the between subject variability for paracetamol.

Aside from the absence of additional benefit, there may also be a higher risk of developing hepatotoxicity when dose is increased beyond 4 g per day. At least, there are conflicting reports on the association of raised aminotransferase concentrations (>3 times upper limits of normal) in healthy adults receiving paracetamol [9,51,52].

## Conclusions

Size and age are important covariates for paracetamol pharmacokinetics, with additional impact of clinical patient characteristics like pregnancy, abdominal and orthopaedic surgery. However, dose individualization based on these covariates would achieve little clinical benefit in the scenarios explored. Since changes in overall paracetamol clearance do not necessary result in proportional changes of the different metabolic elimination routes, further studies on paracetamol metabolism in these specific populations are warranted to identify populations at risk.

## Abbreviations

BOV: Between occasion variability; BSV: Between subject variability; CI: Confidence interval; CL: Clearance; CV: Coefficients of variation; FFM: Free fat mass; HPLC: High pressure liquid chromatography; IV: Intravenous; LBM: Lean body mass; NAPQI: N-acetyl-p-benzoquinone; NFM: Normal fat mass; NONMEM: Non-linear mixed effects modeling; NSAIDS: Non-steroidal anti-inflammatory drugs; OBJ: Objective function change; PC: Prediction corrected; PK: Pharmacokinetics; Q2 and Q3: Intercompartment clearances; TBW: Total body weight; V1: Central volume of distribution; V2 and V3: Peripheral volumes of distribution; PC-VPC: Prediction corrected visual predictive check; WHS: Weight Height Scaler.

## Competing interests

Besides the funding from agencies mentioned below, the authors declare that they have no other competing interests.

## Authors’ contributions

KA took the initiative to contact the different groups and pool the available data and built the pooled data. Data were verified by the other authors (KTO, MVdV, MdM, BJA). BJA performed the population PK analysis. All authors participated in the subsequent interpretation of this analysis, and were involved in drafting the manuscript or revising it critically for important intellectual content. All authors have read and approved the final manuscript.

## Pre-publication history

The pre-publication history for this paper can be accessed here:

http://www.biomedcentral.com/1471-2253/14/77/prepub
